# Agreement of self-reported physician diagnosis of migraine with international classification of headache disorders-II migraine diagnostic criteria in a cross-sectional study of pregnant women

**DOI:** 10.1186/1472-6874-13-50

**Published:** 2013-12-13

**Authors:** Chunfang Qiu, Michelle A Williams, Sheena K Aurora, B Lee Peterlin, Bizu Gelaye, Ihunnaya O Frederick, Daniel A Enquobahrie

**Affiliations:** 1 Center for Perinatal Studies, Swedish Medical Center, 1124 Columbia Street, Suite 750, Seattle, WA 98104, USA; 2 Department of Epidemiology, Harvard School of Public Health, Boston, MA, USA; 3 Department of Neurology, Stanford University, Stanford, California, USA; 4 Johns Hopkins University School of Medicine, Baltimore, MD, USA; 5 Department of Epidemiology, School of Public Health, University of Washington, Seattle, WA, USA

**Keywords:** Migraine, Pregnancy, Diagnosis, ICHD-II, Self-report, Agreement

## Abstract

**Background:**

Migraine, a common chronic-intermittent disorder among reproductive age women, has emerged as a novel risk factor for adverse perinatal outcomes. Diagnostic reliability of self-report of physician-diagnosed migraine has not been investigated in pregnancy cohort studies. We investigated agreement of self-report of physician-diagnosed migraine with the diagnostic criteria promoted by the International Classification of Headache Disorders, 2nd edition (ICHD-II).

**Methods:**

The cross-sectional study was conducted among 500 women who provided information on a detailed migraine questionnaire that allowed us to apply all ICHD-II diagnostic criteria.

**Results:**

Approximately 92% of women reporting a diagnosis of migraine had the diagnosis between the ages of 11 and 40 years (<10 years 6.8%; 11–20 years 38.8%; 21–30 years 42.7%; 31–40 years 10.7%; and >40 years 1.0%). We confirmed self-reported migraine in 81.6% of women when applying the ICHD-II criteria for definitive migraine (63.1%) and probable migraine (18.5%).

**Conclusion:**

There is good agreement between self-reported migraine and ICHD-II-based migraine classification in this pregnancy cohort. We demonstrate the feasibility of using questionnaire-based migraine assessment according to full ICHD-II criteria in epidemiological studies of pregnant women.

## Background

Migraine, a common chronic-intermittent neurovascular headache disorder, is ranked among the world’s twenty most disabling medical conditions by the World Health Organization [1]. Migraine is characterized by episodic severe headache accompanied by autonomic nervous system dysfunction. Women are more commonly affected than men, with reported lifetime prevalence estimates of 16-32% for women and 6-9% for men [2-6]. Migraine risk varies considerably across the life course. The prevalence of migraine in women rises after the average age of menarche, and peaks before the average age of menopause [7]. Thus migraines are most prevalent among women in their childbearing years.

Migraine has emerged as a novel risk factor for adverse perinatal outcomes including hypertensive disorders of pregnancy [8,9], preterm birth [10] and placental abruption [11]. Most prior epidemiologic studies have relied on self-report of physician-diagnosed migraine as a means for classifying pregnant women with a history of migraine. Data from the Women’s Health Study (a study of women aged ≥ 45 years) showed very good agreement between self-reported migraine and ICHD-II-based diagnosed migraine. The investigators confirmed self-reported migraine in > 87% of women when applying the International Classification of Headache Disorders 2004 criteria (ICHD-II) for definitive migraine (71.5%) and probable migraine (16.2%) without aura [12]. We are unaware of studies that investigated diagnostic reliability (commonly measured using inter-rater agreement) of self-report of physician-diagnosed migraine in pregnancy cohort studies.

In a cross-sectional study, we investigated 500 pregnant women who provided information on a detailed migraine questionnaire that allowed us to apply ICHD-II diagnostic criteria, promoted by International Headache Society (IHS), and examined the agreement of self-reported physician diagnosis of migraine with migraine determined using the ICHD-II diagnostic criteria (“golden standard”) in this report.

## Methods

Study participants were pregnant women attending prenatal care at clinics affiliated with Swedish Medical Center in Seattle, Washington and enrolled in the Migraine and Pregnancy Study, a pregnancy cohort study designed to investigate the relationship between migraine, headache symptoms before and during pregnancy, and the risk of preeclampsia [13]. The study population for this report is from the first 500 participants who were enrolled (consecutively) and were interviewed during the period of April 2009 and December 2010. Women were ineligible if they initiated prenatal care after 20 weeks gestation, were younger than 18 years of age, did not speak and read English, did not plan to carry the pregnancy to term, or did not plan to deliver at Swedish Medical Center. Participants completed a questionnaire administered by trained interviewers (supervised by neurologist and maternal fetal medicine clinicians) at enrollment. Participants were asked to provide information pertaining to their medical history, pre-pregnancy weight, general health, pregnancy-related symptoms, socio-demographic, and lifestyle characteristics. The interview included a structured migraine assessment questionnaire (adapted from the deCODE Genetics migraine questionnaire (DMQ3) [14] (Additional file 1) and an assessment of disability associated with headaches experienced before and during pregnancy by Migraine Disability Assessment (MIDAS) Questionnaire [15]. In previous validation study, using a physician-conducted interview as an empirical index of validity, the deCODE Migraine Questionnaire (DMQ3) diagnosed migraine with a sensitivity of 99%, a specificity of 86% and a kappa statistic of 0.89 [16]. The detailed migraine-specific questionnaire contained questions addressing age at migraine onset, physician diagnosis of migraine, family history of migraine, details about migraine attacks and medication used.

Headache classification was determined using the ICHD-II criteria established by the International Headache Society (IHS) [17]. “Definitive Migraine” (IHS category 1.1 or 1.2) was defined by at least five lifetime headache attacks (criterion A) lasting 4–72 hours (criterion B), with at least two of the qualifying pain characteristics [unilateral location (criterion C1), pulsating quality (criterion C2), moderate or severe pain intensity (criterion C3), aggravation by routine physical exertion (criterion C4)]; at least one of the associated symptoms [nausea and/or vomiting (criterion D1), photo/phonophobia (criterion D2)]; and not readily attributable to another central nervous system disorder or head trauma (according to subject self-report) (criterion E). “Probable Migraine” (IHS category 1.6) was designated if all but one of the definitive migraine criteria were fulfilled, excluding headaches attributable to another disorder. Finally, any migraine was defined as the group with either definitive migraine or probable migraine combined.

The procedures used in the study were in agreement with the protocol approved by the Institutional Review Board of Swedish Medical Center (Swedish IRB # 008567). All participants provided written informed consent.

Frequency distributions of sociodemographic, reproductive, medical and behavioral factors among groups defined by ICHD-II (any migraine, no migraine) and the cohort were compared using means (± standard deviation (SD)) for continuous variables and counts and percentages for categorical variables. Bivariate differences in characteristics associated with definitive and probable migraine headaches were determined using Chi-square test (or Fisher’s exact test) for categorical variables and Student’s *t*-test for continuous variables.

The self-reported physician migraine diagnosis was compared with the ICHD-II based diagnosis. The sensitivity and specificity as well as positive predictive value and negative predictive value of the self-reported diagnosis were assessed. Concordance also was determined by estimating the value of Cohen’s kappa coefficient [18]. All analyses were performed using Stata 9.0 (Stata, College Station, TX) statistical analysis software. All reported p-values are two-tailed. The 95% confidence interval (CI) for the prevalence estimate of migraine was determined using previously described methods [19].

## Results

A total of 100 subjects met the ICHD-II criteria for definitive migraine, and 49 additional subjects met the criteria for probable migraine. The lifetime prevalence of definitive migraine was 20.0% (95% CI 16.6-23.8%). When probable migraine was included, the lifetime prevalence of migraine in this population increased to 29.8% (95% CI 25.9-34.0%). However, chronic migraine, defined as headache that occurs 15 or more days a month with headache lasting 4 hours or longer for at least 3 consecutive months in individuals with current or prior diagnosis of migraine, occurred only in 12 women out of 149 ICHD-II diagnosed migraine cases.

Characteristics of participants according to ICHD-II-defined migraine status are presented in Table 1. A positive family history of migraine (defined as report of migraine for at least one of the following relatives: father, mother, sibling, grandparents, children) was reported by 65.1% of participants with migraine and by 35.0% of individuals without migraine. Participants with migraine tended to have higher pre-pregnancy body mass index and were more likely to report a positive history of chronic hypertension.

**Table 1:** Maternal characteristics of study population according to ICHD-II criteria migraine (definitive + probable), April 2009-Dec 2010, Seattle, WA

**Characteristics**	***Cohort***	***ICHD-II criteria for migraine (definitive + probable)***		
	**(N = 500)**	**Yes (N = 149)**	**No (N = 351)**	**p-value**
Maternal Age (years)	33.4 ± 4.2	32.9 ± 4.4	33.6 ± 4.1	0.131
Maternal Age (years)				
< 35	316 (63.2)	102 (68.5)	214 (61.0)	0.112
≥ 35	184 (36.8)	47 (31.5)	137 (39.0)	
Maternal Race/Ethnicity				
Non-Hispanic White	430 (86.0)	126 (84.6)	304 (86.6)	0.593
African American	8 (1.6)	4 (2.7)	4 (1.1)	
Other	57 (11.4)	18 (12.1)	39 (11.1)	
Missing	5 (1.0)	1 (0.7)	4 (1.1)	
Annual Household Income ($)				
<50,000	20 (4.0)	6 (4.0)	14 (4.0)	0.061
50,000-69,000	33 (6.6)	13 (8.7)	20 (5.7)	
≥ 70,000	428 (85.6)	129 (86.6)	299 (85.2)	
Missing	19 (3.8)	1 (0.7)	18 (5.1)	
Single Marital Status	48 (9.6)	19 (12.8)	29 (8.3)	0.119
Nulliparous	261 (52.2)	81 (54.4)	180 (51.3)	0.528
Unplanned Pregnancy	72 (14.4)	24 (16.1)	48 (13.7)	0.479
Cigarette Smoker during early pregnancy	95 (19.0)	30 (20.1)	65 (18.5)	0.674
Family History of Headache/Migraine	220 (44.0)	97 (65.1)	123 (35.0)	<0.001
History of Chronic Hypertension	11 (2.2)	7 (4.7)	4 (1.1)	0.020
Pre-Pregnancy Body Mass Index (kg/m^2^)	23.5 ± 4.7	24.7 ± 5.4	23.0 ± 4.3	<0.001
Pre-Pregnancy Body Mass Index (kg/m^2^)				
<18.5	24 (4.8)	5 (3.4)	19 (5.4)	0.012
18.5-24.9	340 (68.0)	89 (59.7)	251 (71.5)	
25.0-29.9	92 (18.4)	34 (22.8)	58 (16.5)	
≥ 30.0	42 (8.4)	20 (13.4)	22 (6.3)	
Missing	2 (0.4)	1 (0.7)	1 (0.3)	

Data in mean ± standard deviation (SD) or number (%).

**Figure 1:**
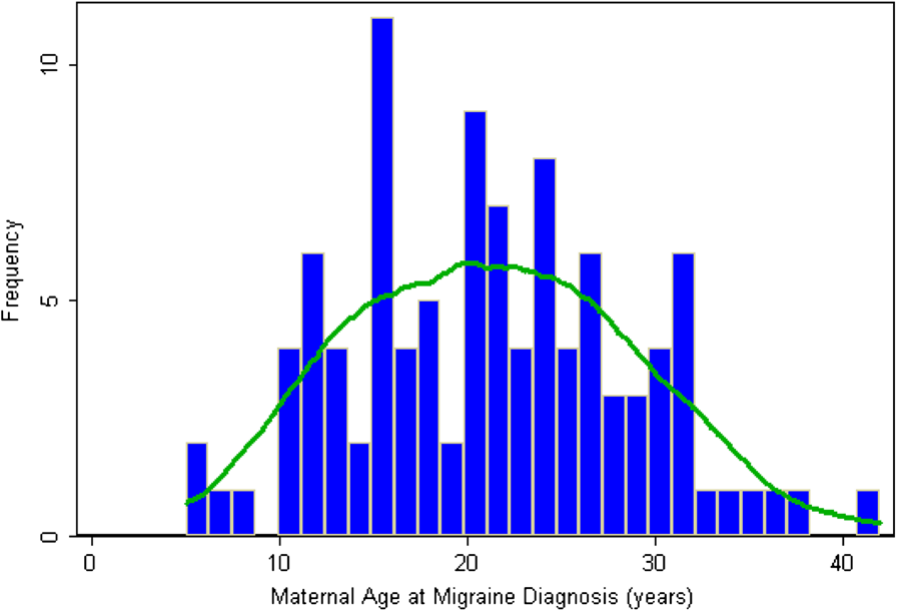
Frequency distribution and Kernel density plot of maternal age at self-reported migraine diagnosis.

We confirmed self-reported migraine in 81.6% of women when applying the ICHD-II criteria for definitive migraine (63.1%) and probable migraine (18.5%) (Table 2). Overall, when compared to the ICHD-II diagnosis, the self-reported physician diagnosis had a sensitivity of 56.8% (95% CI 48.7%-64.5%); a specificity of 94.6% (95% CI 91.7%-96.5%) and positive predictive value of 81.6% (95% CI 73.0%-87.9%), as well as negative predictive value of 83.8% (95% CI 79.8%-87.1%). Cohen’s kappa coefficient was 0.56. We did not observe differences in Choen’s kappa coefficients when analyses were stratified by parity (i.e., nulliparous vs. multiparous). Characteristics such as nausea and/or vomiting and photophobia were far more prevalent among women who reported having a physician diagnosis of migraine as compared with their counterparts without such a diagnosis. Approximately 92% of women reporting a diagnosis of migraine had the diagnosis between the ages of 11 and 40 years (<10 years 6.8%; 11–20 years 38.8%; 21–30 years 42.7%; 31–40 years 10.7%; and >40 years 1.0%) (Figure 1).

**Table 2:** Summary of self-reported migraine and ICHD-II classified migraine

**Characteristics**	**Self-reported migraine**	
	**Yes**	**No**
	**(N = 103)**	**(N = 395)**
ICHD-II Diagnostic Criteria: Migraine		
No	19 (18.5)	331 (83.8)
Yes	84 (81.6)	64 (16.2)
*ICHD-II criteria migraine (probable)*	19 (18.5)	29 (7.3)
*ICHD-II criteria migraine (definitive)*	65 (63.1)	35 (8.9)
Lifetime number of headache/migraine attacks ≥5 (criterion A)	95 (92.2)	94 (23.8)
Attacks lasting 4–72 hours (criterion B)	80 (77.7)	73 (18.5)
Unilateral headache location (criterion C1)	59 (57.3)	43 (10.9)
Pulsating pain quality (criterion C2)	62 (60.2)	60 (15.2)
Moderate or severe pain intensity (criterion C3)	95 (92.2)	101 (25.6)
Aggravation by routine physical activity (criterion C4)	72 (69.9)	70 (17.7)
Nausea and/or vomiting (criterion D1)	80 (77.7)	64 (16.2)
Photophobia	91 (88.4)	90 (22.8)
Phonophobia	86 (83.5)	79 (20.0)
Photo- and phonophobia (criterion D2)	82 (79.6)	66 (16.7)

2 subjects missing self-reported migraine information.

Data in number (%).

*ICHD-II*, International Classification of Headache Disorders, version 2 (ICHD-II).

## Discussion

Our study finding of a high prevalence (29.8%) of migraine in this cohort of pregnant women of reproductive age is consistent with prior literature [3-5]. For example, the American Migraine Prevalence and Prevention (AMPP) Study found that the highest 1-year period prevalence was observed among those ages 18–59. In that cohort, 17.1% of women met diagnostic criteria for definitive migraine; and additional 5.1% met diagnostic criteria for probable migraine [5]. Our study also showed good agreement between self-reported migraine and ICHD-II-based migraine classification. Our results were consistent with reports by Schurks et al., who reported good agreement between self-reported migraine and ICHD-II defined migraine in a cohort of non-pregnant women [12]. Since migraine diagnosis relies on the presence of certain symptoms as well as associated features, information that can easily be obtained using questionnaires, the agreement of such self-reports with the ICHD-II criteria is crucial for studying migraine-specific characteristics in population-based epidemiological studies.

The strengths of our study include the relatively large number of women with information from the detailed standardized questionnaire allowing us to apply all ICHD-II criteria for migraine, and the opportunity to compare with self-reported “physician-diagnosed” migraine information. To our best knowledge, this is the first study that investigated the agreement of self-reported migraine with ICHD-II diagnosis in a pregnancy cohort. Pregnancy is a unique condition. During the first trimester, women experience increased physiological and psychological changes such as hormonal surge and increased blood volume. Lack of sleep, low blood sugar, dehydration and possible caffeine withdrawal could also contribute to the occurrence of migraine attacks. The headache may be further aggravated by stress [20,21]. In addition, there is a heightened cautiousness among women with regards to using medication during early pregnancy to prevent or treat their headaches. Concurrently, most studies on migraine in pregnancy detected a decrease in frequency of reported migraine attacks and pain intensity in the 2nd and 3rd trimesters as hormone levels stabilize [22-24].

Some important limitations must be considered when interpreting the results of our study. First, our ICHD-II-diagnosed migraine is based on questionnaires administered by trained interviewers instead of the gold standard of physician examination. However, it is not feasible to take implement full scale physical examinations with medical histories in large epidemiological studies, making our approach a practical alternative. In this study, we observed good agreement between ICHD-II diagnosed migraine and self-reported “physician diagnosed” migraine. Secondly, misclassification of headache type is possible. The questionnaire did not ask women to describe multiple headache types, and it is presumed that subjects described the headache episodes most burdensome to them. Misunderstanding one or more questions on the migraine-specific questionnaire may also lead to a misclassification according to the ICHD-II criteria. Third, caution must be taken that a diagnosis made during pregnancy may be attributable to transient changes in the characters of primitive headaches [25]; however, in the current study, 93.3% of ICHD-II defined migraine patients reported that their headache attacks started more than one year before the interview, hence mitigating concern of misclassification in this case. Finally, in this study, only a small minority of women with migraine were classified as having chronic migraine *per se*. Recall bias is possible although migraines are usually dramatic events well remembered by the affected persons.

## Conclusions

There is good agreement between self-reported physician-diagnosed migraine and ICHD-II-based migraine classification in this pregnancy cohort. Our findings demonstrate the feasibility of using questionnaire-based migraine assessment according to full ICHD-II criteria in epidemiological studies of pregnant women.

## Abbreviations

ICHD-II: The International Classification of Headache Disorders 2004 criteria, version II; IHS: International Headache Society; SD: Standard deviation; 95% CI: 95% confidence interval; AMPP: American Migraine Prevalence and Prevention.

## Competing interests

The authors declare that they have no competing interests.

## Authors’ contributions

MAW acquired funding for the study and developed the study design. SKA and BLP helped develop the study design. IOF monitored the data collection. CQ, DAE and MAW developed the analytical plan; CQ completed the statistical analysis. CQ, DAE, MAW drafted the manuscript. All authors edited the manuscript. All authors read and approved the final manuscript.

## Pre-publication history

The pre-publication history for this paper can be accessed here:

http://www.biomedcentral.com/1472-6874/13/50/prepub

## Supplementary Material

Additional file 1 The deCODE Genetics migraine questionnaire (DMQ3) Click here for file
