# Supplementary material for: Agreement of self-reported physician diagnosis of migraine with international classification of headache disorders-II migraine diagnostic criteria in a cross-sectional study of pregnant women
Source: BMC Womens Health. 2013 Dec 13;13:50. doi: 10.1186/1472-6874-13-50 (PMC3878724; doi:10.1186/1472-6874-13-50)
Supplement: Additional file 1 — The deCODE Genetics migraine questionnaire (DMQ3) [file 1472-6874-13-50-S1.pdf]

Questionnaire

The Genetics of Migraine

Gretar Guðmundsson, MD
Maria G. Hrafnsdottir, MD
John Benedikz, MD

IN RESEARCH COLLABORATION WITH

deCODE genetics

Date: ____I ____I--____I ____I-- ____I ____I
d d m m y y

Name: __

Unique ID: ___I ___I ___I ___I ___I ___I -- ___I ___I ___I ___I

The information you provide on the following pages will be processed according to the rules of the Icelandic Privacy Protection Commission

---

The following questionnaire contains questions on health history and lifestyle that concerns the disease **migraine** and may influence its progress.

Your answers may provide worthy information on the interaction of lifestyle, environment and genetic factors in the development of this disease.

You are free to skip particular questions or the questionnaire as a whole. If any of the questions are unclear, or you need more information on the questionnaire, research personnel will be happy to provide you with the information you need. Please contact: The Genetic Research Service Center in Noatun, tel. 520-2800.

Instructions:
Using a dark pencil (#2), please fill in the circle for your chosen answer as follows:

Example (if your answer is ”no”): ● No O Yes

deCODE genetics

1. Do you currently, or have you ever, experienced headache attacks?

O no O yes

If the answer is no, please answer next question 29 on page 12

2. How long ago did these headache attacks start?

O less than a month
O 1 – 12 months
O longer than 12 months (one year)

3. When was the last time you had a headache attack?

O less than a year ago
O 1 – 5 years ago
O more than 5 years ago

4. How old were you when the headache attacks started?

O younger than 10 years old
O 10-19 yrs
O 20-29 yrs
O 30-39 yrs
O 40-49 yrs
O 50-60 yrs
O older than 60 years old

5. Have you been diagnosed with migraine?

O no O yes

5.1. How old were you when you were diagnosed with migraine?

O younger than 10 years of age
O 10-19 yrs
O 20-29 yrs
O 30-39 yrs
O 40-49 yrs
O 50-60 yrs
O older than 60 years of age

6. How many attacks of headache have you had?

O less than 2 attacks O 2 - 4 attacks O 5 or more attacks

7. For how long do the headache attacks usually last?

O less than 4 hours
O 4 – 72 hours
O more than 72 hours (3 days)

8. Have you noticed any change in how you feel in the last day or two before a headache attack starts?

O no O yes

8.1. **If yes, which changes?** ***(please mark all relevant options)***

O unusually good mental or physical feeling
O an urge to start a big project or to start many things at the same time
O a strong craving for certain type of food
O reduced or enhanced libido
O worse physical or mental feeling than circumstances allow
O other, what? ____

9. Do you get one or more of the listed symptoms (called aura) shortly <u>before</u> headache onset?

**Visual disturbances** (e.g. flares or zigzag pattern, a blind spot, or spots before the eyes)	O no	O yes
**Sensory disturbances** (e.g. numbness that spreads slowly over the face or one side of the body, partly or completely)	O no	O yes
**Speech disturbances** (e.g. difficulties in finding words, forming sentences or understanding spoken words)	O no	O yes
**Decreased strength or paralysis** in one side of the body	O no	O yes
**Some other form of aura**, what? ______________________________________	O no	O yes

If you answered no to all items in question 9, please turn to question 12

9.1. If you have experienced aura before the headache onset, how many times has it happened?

O Once O Twice or more O Always

9.2. In your own words, please describe your aura symptoms and how they begin and develop.

____

____

____

____

____

____

____

10. If you experience any of the aura symptoms listed in question 9, how long time do they last? ***(please mark all relevant options)***

Visual disturbances:	O less than 60 min.	O around 60 min.	O longer than 60 min.
Sensory disturbances:	O less than 60 min.	O around 60 min.	O longer than 60 min.
Speech disturbances:	O less than 60 min.	O around 60 min.	O longer than 60 min.
Strength reduction/paralysis:	O less than 60 min.	O around 60 min.	O longer than 60 min.
Altogether:	O less than 60 min. (1 hour)	O 60-240 min. (1 – 4 hrs)	O longer than 240 min. (4 hrs)

11. How does the headache usually begin? ***(please mark only one item)***

O during the aura?
O within one hour from the disappearance of the aura?
O more than an hour after the disappearance of the aura?

12. How do the headache attacks usually begin? (If your headache begins in the forehead, please indicate on which side of the forehead)

O both sides of the head
O always on the right side of the head
O always on the left side of the head
O alternatively on the left or the right side of the head, depending on the attacks

13. Is the pain usually in the back of your head?

O no O yes

14. Do you associate your headache attacks with muscle soreness in your neck or shoulders?

O no O yes

15. Do you associate your headache attacks with anything you eat or drink?

O no O yes

15.1. If yes, which food or beverages?

O chocolate
O cheese
O citrus fruit (e.g. oranges or lemons)
O bananas
O other fruit
O monosodium glutamate (MSG)
O red wine
O other alcoholic beverages than red wine
O any other, what? ___________________________________

16. Does any of the listed options describe your headaches (either in some or all attacks)?

moderate or severe headaches that disturb or inhibit your daily activities	O no	O yes
pulsating headaches (in rhythm with your pulse/heartbeat)	O no	O yes
headaches that worsen during exertion, e.g. when walking stairs	O no	O yes
headaches associated with nausea or vomiting	O no	O yes
headaches worsen in bright light	O no	O yes
headaches worsen in noise	O no	O yes

17. Have you been diagnosed with any of the listed CNS diseases or physical conditions, that are considered to be the cause of your headache attacks? (please, mark only items that are considered to cause your headache attacks)

O long-term headaches related to trauma or following trauma
O cerebral occlusion due to blood clot in the brain
O short-term interruption of blood circulation to the brain (transient ischemic attack or TIA)
O cerebral hemorrhage
O meningeal hemorrhage
O malformation or flaw in the vascular system of the head
O cerebral aneurysm (ruptured or not)
O inflammation in cerebral blood vessel walls or in blood vessels elsewhere in the body
O another blood vessel disease of the head
O a rupture of jugular veins
O unexplained pain from jugular veins
O headaches following blood vessel operation in the head
O a cerebral occlusion in the vascular system of the head
O hypertension
O a hormone (adrenaline, nor-adrenaline)-producing tumor (pheochromocytoma)
O pre-eclampsia (toxemia during pregnancy)
O increased brain and spinal fluid pressure
O too low spinal fluid pressure or a spinal fluid leak
O headaches that started following a lumbar puncture
O headaches that started following an infection of the tissues within the scull (encephalitis or meningitis)
O inflammatory diseases within the scull other than infections (e.g. lupus erythematosis or sarcoidosis)
O headaches that started after receiving spinal fluid medication
O headaches that started in relation to a brain tumor
O headaches that started because of nitrates (e.g. nitroglycerin tablets)
O headaches that are related to long term use of ergotamine containing medications (e.g. Anervan, Caffergot, etc.), painkillers, or headaches due to withdrawal of these medications
O headaches because of caffeine withdrawal
O headaches because of nicotine withdrawal
O other reasons? What ____

17.1. If you have answered yes to anything in question 17, are these the headaches you have in mind when answering this questionnaire?

O no O yes

Males answer next question 20

18. Do you associate your headache attacks with the menstrual cycle?

O no O yes

19. Do you get headaches when using hormonal medications (e.g. when taking a birth control pill or menopausal hormone replacement therapy)?

O no O yes O do not use hormonal medicine

20. Are your headache attacks considered to be caused by any of the following?

hypoglycemia (low blood sugar)	O no	O yes
cervical vertebrae problems, e.g. trauma or joint degeneration	O no	O yes
eye diseases, e.g. glaucoma, astigmatism, myopia, or hypermetropia	O no	O yes
a disease in ears, nose, sinuses, mouth, teeth, or the temporomandibular joint(s)	O no	O yes
common illnesses such as the flu or a cold	O no	O yes
sleep apnea	O no	O yes

21. Do you know if any of the following were true about you as a child?

car or motion sickness	O no	O yes
unexplained instances of high fever	O no	O yes
stomach aches (other than infant colic)	O no	O yes
recurrent vomiting without other symptoms	O no	O yes
dizzy-spells	O no	O yes
headaches	O no	O yes

If nothing of the above was true, please answer next question 22

21.1. Was your illness as a child treated in any way?

O no O yes, in what way? (if medication, what medicine?)

____
____
____

22. Have you had any of the following tests because of headaches?

X-ray of the head or neck	O no	O yes
a computerized tomography (CT) scan of your head	O no	O yes
a magnetic resonance image (MRI) of the head	O no	O yes
an electroencephalogram (EEG)	O no	O yes
a blood test	O no	O yes
other tests ____________________	O no	O yes

If the answer to all this was negative, please answer next question 23

22.1. If any tests have been done because of the headaches, what were the results?

O normal
O abnormal, how ____________________
____
____

23. Do any relatives of yours get headache attacks or have migraine?

O no O yes

23.1. If *yes*, which relatives? *(please mark all relevant)*

O child/children
O brothers or sisters
O father
O mother
O any relatives in your mother's family
O any relatives in your father's family

24. Are you:

O right handed? O left handed?

25. Do you use a medication on a regular basis to prevent frequent headaches?

O no O yes

25.1. If *yes*, which medication do you currently use to prevent headaches?

Name of medicine:	Strength (mg)	Taken how many times a day?
1) ____________	______	______
2) ____________	______	______
3) ____________	______	______
4) ____________	______	______
5) ____________	______	______

26. Do you use a medication to treat your headache when it has started or when you sense a coming headache?

O no O yes

26.1. If *yes*, which medication do you currently use to treat your headache when it has started or when you sense a coming headache?

Name of medicine:	Strength (mg)
1) ____________	______
2) ____________	______
3) ____________	______
4) ____________	______
5) ____________	______

27. Have you ever <u>successfully</u> used <u>methods other than medication</u> to treat or to prevent headache attacks?

O no O yes

27.1. If *yes*, what method have you used to treat or prevent headache attacks?

acupuncture	O no	O yes
electrical stimulation	O no	O yes
hot or cold compresses	O no	O yes
massage	O no	O yes
other, what? ___________________________________	O no	O yes

28. Guidelines: Please answer the following questions about ALL your headaches you have had over the last 3 months. Write your answer in the field next to each question. Write zero if you did not do the activity in the last 3 months. If necessary, you can refer to the calendar on the next page.

28.1. On how many days in the last 3 months did you miss work or school because of your headache? I__I__I days

28.2. How many days in the last 3 months was your productivity at work or school reduced by half or more because of your headaches? (Do not include days you counted in previous question.) I__I__I days

28.3. On how many days in the last 3 months did you not do household work because of your headache? I__I__I days

28.4. How many days in the last 3 months was your productivity in household work reduced by half or more because of your headaches? (Do not include days you counted in previous question.) I__I__I days

28.5. On how many days in the last 3 months did you miss family, social or leisure activities because of your headaches? I__I__I days

28.A. On how many days in the last 3 months did you have a headache? (If a headache lasted more than 1 day, count each day.) I__I__I days

28.B. On a scale of 0 – 10, on average how painful were these headaches? (where 0 = no pain at all, and 10 = pain as bad it can be) I__I__I

Calendar for year 2003

January

Su	Mo	Tu	We	Th	Fr	Sa
			1	2	3	4
5	6	7	8	9	10	11
12	13	14	15	16	17	18
19	20	21	22	23	24	25
26	27	28	29	30	31	

February

Su	Mo	Tu	We	Th	Fr	Sa
						1
2	3	4	5	6	7	8
9	10	11	12	13	14	15
16	17	18	19	20	21	22
23	24	25	26	27	28	

March

Su	Mo	Tu	We	Th	Fr	Sa
						1
2	3	4	5	6	7	8
9	10	11	12	13	14	15
16	17	18	19	20	21	22
23	24	25	26	27	28	29
30	31					

April

Su	Mo	Tu	We	Th	Fr	Sa
		1	2	3	4	5
6	7	8	9	10	11	12
13	14	15	16	17	18	19
20	21	22	23	24	25	26
27	28	29	30			

May

Su	Mo	Tu	We	Th	Fr	Sa
				1	2	3
4	5	6	7	8	9	10
11	12	13	14	15	16	17
18	19	20	21	22	23	24
25	26	27	28	29	30	31

June

Su	Mo	Tu	We	Th	Fr	Sa
1	2	3	4	5	6	7
8	9	10	11	12	13	14
15	16	17	18	19	20	21
22	23	24	25	26	27	28
29	30					

July

Su	Mo	Tu	We	Th	Fr	Sa
		1	2	3	4	5
6	7	8	9	10	11	12
13	14	15	16	17	18	19
20	21	22	23	24	25	26
27	28	29	30	31		

August

Su	Mo	Tu	We	Th	Fr	Sa
					1	2
3	4	5	6	7	8	9
10	11	12	13	14	15	16
17	18	19	20	21	22	23
24	25	26	27	28	29	30
31						

September

Su	Mo	Tu	We	Th	Fr	Sa
	1	2	3	4	5	6
7	8	9	10	11	12	13
14	15	16	17	18	19	20
21	22	23	24	25	26	27
28	29	30				

October

Su	Mo	Tu	We	Th	Fr	Sa
			1	2	3	4
5	6	7	8	9	10	11
12	13	14	15	16	17	18
19	20	21	22	23	24	25
26	27	28	29	30	31	

November

Su	Mo	Tu	We	Th	Fr	Sa
						1
2	3	4	5	6	7	8
9	10	11	12	13	14	15
16	17	18	19	20	21	22
23	24	25	26	27	28	29
30						

December

Su	Mo	Tu	We	Th	Fr	Sa
	1	2	3	4	5	6
7	8	9	10	11	12	13
14	15	16	17	18	19	20
21	22	23	24	25	26	27
28	29	30	31			

QUESTIONS ON OTHER DISEASES

29. Have you been diagnosed by a physician or a psychologist with any of the following diseases?

O epilepsy
O stroke
O allergy
O asthma
O hereditary hand tremor (essential tremor)
O any other diseases (migraine not included): ____
____

QUESTIONS ON TOBACCO USAGE

30. Have you ever smoked or used tobacco for as long as a year?

O yes   O no (if *no,* please answer next question **31 on page 14**)

30.1. How old were you when you first started smoking or using tobacco?

O younger than 11 years old	O 12 years old	O 13 years old
O 14 years old	O 15 years old	O 16 years old
O 17 years old	O 18 years old	O 19 years old
O 20 – 21 years old	O 22-24 years old	O 25-29 years old
O 30-34 years old	O 35 years or older	

30.2. Have you currently stopped smoking / using tobacco?

O no   O yes

If *yes*, how old were you when you last quit smoking / using tobacco?

**O** Younger than 15 yrs	**O** 15-19 yrs	**O** 20-24 yrs	**O** 25-29 yrs	**O** 30-31 yrs
**O** 31-32 yrs	**O** 32-33 yrs	**O** 33-34 yrs	**O** 35-36 yrs	**O** 37-38 yrs
**O** 39-40 yrs	**O** 41-42 yrs	**O** 43-44 yrs	**O** 45-46 yrs	**O** 47-48 yrs
**O** 49-50 yrs	**O** 51-52 yrs	**O** 53-54 yrs	**O** 55-56 yrs	**O** 57-58 yrs
**O** 59-60 yrs	**O** 61-62 yrs	**O** 63-64 yrs	**O** 65-66 yrs	**O** 67-68 yrs
**O** 69-70 yrs	**O** 71-72 yrs	**O** 73-74 yrs	**O** 75-76 yrs	**O** 77-78 yrs
**O** 79-80 yrs	**O** 80 or older			

30.3. How many cigarettes a day do / did you smoke on average (on most days)?
(1 pack = 20 cigarettes)

(please mark what applied to your smoking on most days)

O Never smoked cigarettes

O 1 – 2	O 3 – 4	O 5 – 6	O 7 – 8	O 9 – 10
O 11 – 12	O 13 – 14	O 15 – 16	O 17 – 18	O 19 – 20
O 21 – 22	O 23 – 24	O 25 – 29	O 30 – 34	O 35 – 44

O more than 45 cigarettes   O only smoke(d) socially (irregularly)

How many cigarillos do / did you smoke on an average day?

O Never smoked cigarillos

O 1 – 2	O 3 -4	O 5 – 6	O 7 – 8	O 9 – 10
O 11 – 12	O 13 – 14	O 15 – 16	O 17 – 18	O 19 – 20

O 21 – 22   O more than 22 cigarillos a day

O only smoke(d) socially (irregularly)

How many cigars do / did you smoke in an average week?

O Never smoked cigars

O 1 – 2	O 3 – 4	O 5 – 6	O 7 – 8	O 9 – 10
O 11 – 12	O 13 – 14	O 15 – 16	O 17 – 18	O 19 – 20
O 21 – 24	O 25 – 29	O 30 – 34	O 35 – 39	O 40 – 44
O 45 – 49	O 50 – 54	O 55 – 59	O 60 – 64	O 65 – 69

O 70 or more cigars / week   O only smoke(d) socially (irregularly)

How many envelopes of pipe tobacco do / did you smoke in an average week?

O Never smoked pipe tobacco

O less than ½ envelope   O ½-1 envelope

O more than 1 envelope but less than 1½

O 1½-2 envelopes   O more than 2 envelopes but less than 2½

O 2½-3 envelopes   O more than 3 envelopes

How many cans of snuff do / did you use in an average week?

O Never used snuff

O less than ½ a can O ½ - 1 can O more than 1 can but less than 1½

O 1 ½ - 2 cans O more than 2 cans but less than 2½ O 2½ - 3 cans

O more than 3 cans

Do / did you use snuff by nose or mouth?

O Only by nose O Only by mouth O Both by nose and mouth

How many cans of chewing tobacco do / did you use in an average week?

O Never used chewing tobacco

O less than ½ a can O ½ - 1 can O more than 1 can but less than 1½

O 1 ½ - 2 cans O more than 2 cans but less than 2½ O 2½ - 3 cans

O more than 3 cans

31. Do you, or have you lived in a home where you were exposed to tobacco smoke?

O no O yes

Number of years in childhood (0-18 years of age) that you were exposed to tobacco smoke in your home:

O 0 – 1 year O 2 – 5 years O 6 – 9 years O 10 – 13 years O 14 – 18 years

Number of years after age 18 that you were exposed to tobacco smoke in your home:

O 0 – 1 year O 2 – 5 years O 6 – 9 years O 10 – 13 years O more than 14 years

32. Have you ever used alcohol?

O no (if *no*, please answer next question **33 on page 16**) O yes

32.1. If *yes,* how much alcohol did you drink, in an average week for the past year? If you *have stopped using alcohol* which of the following does best apply to your weekly consumption of alcohol in the last 12 months before you stopped?

Note! 1 drink = 1 beer = 1 glass of wine = 1 shot of cordials or hard liquor

O None or less than one drink a week

O 1-6 drinks a week

O 7-14 drinks a week

O 15-28 drinks a week

O more than 28 drinks a week

32.2. For the past 12 months, on how many days a week on average did you drink alcohol? If *you have stopped drinking alcohol* on how many days a week on average did you drink alcohol for the past 12 months before you stopped?

O 0 – 1 day a week (less than one day a week) O 2 days a week

O 3 days a week O 4 days a week O 5 days a week

O 6 days a week O 7 days a week

33. How much physical exercise is involved in the job you have had for most of your life?

O very light exertion

- involves sitting during most of the day (e.g. office job)

O rather light exertion

- involves standing most of the day, seldom lifting loads or carrying, e.g. washing up, service

O medium exertion

- involves lifting or carrying light loads, walking stairs or slight slopes, e.g. walking around, lifting or carrying more than 5 kg, housecleaning, babysitting

O considerable exertion

- involves physical labor that causes shortness of breath, e.g. laborious service or industry work

O heavy exertion

- involves physically straining work, carrying heavy loads, e.g. concrete work, shoveling, lifting more than 25 kg

34. Do you currently participate in sports or regular physical exercise?

O no O yes O don’t know

If yes, how many hours a week on average did you exercise in the past year?

O less than 1 hour a week

O more than 1, less than 2 hours

O more than 2, less than 3 hours

O more than 3, less than 4 hours

O more than 4, less than 5 hours

O more than 5, less than 6 hours

O more than 6, less than 7 hours

O 7 hours or more a week

O don’t know

35. Have you participated regularly in sports or other physical exercise after reaching 20 years of age?

O no O yes O don’t know

35.1. If *yes*, at what age did you participate regularly in sports or other physical exercise? *(please mark all relevant)*

O 20-29 years of age O 30-39 years of age O 40-49 years of age

O 50-59 years of age O 60-69 years of age O 70-79 years of age

O 80 years or older

Thank you for your participation
